# Altered Functional Connectivity Strength at Rest in Medication-Free Obsessive-Compulsive Disorder

**DOI:** 10.1155/2021/3741104

**Published:** 2021-09-08

**Authors:** Dan Lv, Yangpan Ou, Yuhua Wang, Jidong Ma, Chuang Zhan, Ru Yang, Yunhui Chen, Tinghuizi Shang, Cuicui Jia, Lei Sun, Guangfeng Zhang, Zhenghai Sun, Jinyang Li, Xiaoping Wang, Wenbin Guo, Ping Li

**Affiliations:** ^1^Department of Psychiatry, Qiqihar Medical University, Qiqihar, China; ^2^National Clinical Research Center for Mental Disorders and Department of Psychiatry, The Second Xiangya Hospital of Central South University, Changsha, China; ^3^Department of Psychiatry, Baiyupao Psychiatric Hospital of Harbin, Harbin, China; ^4^Department of Radiology, The Second Xiangya Hospital of Central South University, Changsha, China; ^5^Department of Radiology, The Third Affiliated Hospital of Qiqihar Medical University, Qiqihar, China

## Abstract

**Background:**

Previous studies explored the whole-brain functional connectome using the degree approach in patients with obsessive-compulsive disorder (OCD). However, whether the altered degree values can be used to discriminate OCD from healthy controls (HCs) remains unclear.

**Methods:**

A total of 40 medication-free patients with OCD and 38 HCs underwent a resting-state functional magnetic resonance imaging (rs-fMRI) scan. Data were analyzed with the degree approach and a support vector machine (SVM) classifier.

**Results:**

Patients with OCD showed increased degree values in the left thalamus and left cerebellum Crus I and decreased degree values in the left dorsolateral prefrontal cortex, right precuneus, and left postcentral gyrus. SVM classification analysis indicated that the increased degree value in the left thalamus is a marker of OCD, with an acceptable accuracy of 88.46%, sensitivity of 87.50%, and specificity of 89.47%.

**Conclusion:**

Altered degree values within and outside the cortical-striatal-thalamic-cortical (CSTC) circuit may cocontribute to the pathophysiology of OCD. Increased degree values of the left thalamus can be used as a future marker for OCD understanding-classification.

## 1. Introduction

Obsessive-compulsive disorder (OCD) is defined as a combination of intrusive thoughts (obsessions) and repetitive behaviors (compulsions), which affects social and occupational functions and imposes an economic burden on patients and their families [1, 2]. Although the pathophysiological mechanism of OCD remains unclear, neuroimaging studies have highlighted abnormalities in the cortical-striatal-thalamic-cortical (CSTC) circuit, including the anterior cingulate cortex, orbitofrontal cortex (OFC), dorsolateral prefrontal cortex (DLPFC), thalamus, and striatum [3–6]. For example, increased and decreased levels of gray matter volumes in the left OFC and striatum and increased regional homogeneity (ReHo) and global brain functional connectivity (FC) in the lateral OFC and DLPFC were discovered at rest in OCD [7, 8]. Moreover, abnormal white matter within the CSTC circuit is associated with the clinical symptoms of OCD [9].

FC patterns at a resting state display a temporal correlation and provide the communication and interaction between spatially separated brain regions [10]. Previous studies applied a region-of-interest (ROI) approach to investigate the FC alterations in given brain regions at rest in OCD with inconsistent results [11–13]. The ROI analysis estimates the strength and significant series of correlations between a given brain region and all other brain regions. However, it may miss the crucial brain regions related to the pathophysiological mechanism of OCD [14]. The voxel-wise degree analysis which is data-driven and high-resolution can be used to explore the pathophysiology of OCD to remedy this shortage. Degree analysis calculates the number of instantaneous FC of each voxel with other voxels in the whole brain rather than the given ROIs [15]. Compared to other FC methods, the advantage of degree analysis is obtaining FC throughout the whole brain in an unbiased way. Therefore, it can be used as an important index for evaluating the FC strength [15]. For this background, the degree analysis approach was used to investigate the pathophysiological mechanism of OCD from the FC alterations throughout the whole brain at rest in the present study. Previous studies have delineated the degree values from FC in schizophrenia [16] and Alzheimer's disease [17] to physical connectivity in patients with major depression [18], alcohol dependence [19], and schizophrenia [20]. In addition, increased degree values in the OFC and basal ganglia were found at rest in OCD [21], and changes in the degree of the right ventral frontal cortex were related to the alleviation of OCD symptoms. Furthermore, a decreased degree of the bilateral superficial amygdala can be used in predicting the effect of cognitive behavior therapy in OCD [22]. Although these studies used the degree approach to explore the whole-brain functional connectome in patients with OCD, whether the altered degree values can be used in discriminating OCD from healthy controls (HCs) remains unclear.

A support vector machine (SVM) is the most commonly used pattern of recognition algorithm in neuroimaging research, providing optimally distinguished categories by establishing a decision function or hyperplane based on well-defined datasets. Then, it utilizes the generated decision function or hyperplane to forecast a new observation belonging to the predefined group [23]. In the SVM analysis, feature selection is the key step to reduce the redundancy and to select meaningful features from the original feature sets [24]. The remaining meaningful features are integrated into a specific classifier via an embedded manner for SVM training [24]. Classification is the approach of classifying the given input by training with an appropriate classifier [25]. Many researchers suggested that SVM is an effective method to construct classifiers [25, 26]. Therefore, our present research applied the SVM method to detect whether abnormal degree values can be used in classifying patients with OCD from HCs.

In the current study, we compared the whole-brain functional connectome at rest in OCD and HCs with the degree approach. Moreover, SVM was used in determining whether abnormal degree values could be used in discriminating OCD from HCs. Based on previous studies, we hypothesized that patients with OCD would show altered degree values in the CSTC circuit at rest, and the altered degree values would be correlated with the clinical symptoms of OCD and could be used in differentiating OCD from HCs.

## 2. Materials and Methods

### 2.1. Subjects

We enrolled 40 medication-free patients with OCD from the Fourth Affiliated Hospital of Qiqihar Medical University and Qiqihar Mental Health Center, China, and 38 HCs from the community. The two groups were matched for gender, age, and education level. Diagnoses of OCD were confirmed with the Structured Clinical Interview for DMS-IV (SCID) (patient version). HCs were screened with the nonpatient version of SCID. The severity of OCD, anxiety, and depressive symptoms was evaluated using the Yale-Brown Obsessive-Compulsive Scale (Y-BOCS), Hamilton Anxiety Rating Scale (HAMA), and 17-item Hamilton Rating Scale for Depression (HAMD), respectively. OCD patients with Y-BOCS total scores of greater than 16 and HAMD scores of less than 18 were considered eligible for the study. All the patients were free of any medication for at least 4 weeks before the brain image acquisition (18 patients were drug naïve, whereas 22 had a history of antiobsessive, antidepressant, or antipsychotic medication). The inclusion criteria were as follows: (1) 16-50 years of age; (2) Han Chinese, right-handed; (3) no acute physical disease and psychiatric or neurological illness; (4) no alcohol or drug dependence; (5) no contraindications for the MRI scan; and (6) no movement distance of more than 2 mm nor rotation angle of more than 2°. HCs with first-degree relatives suffering from any psychiatric disorder were excluded.

The current study was approved by the Medical Ethics Committee of Qiqihar Medical University. The subjects signed written informed consent forms after being informed of the study procedures.

### 2.2. Image Acquisition and Preprocessing

All imaging data were acquired using a 3.0-Tesla GE 750 Signa-HDX scanner at the Third Affiliated Hospital of Qiqihar Medical University, China. None of the subjects had clinically significant brain structural damage. The resting-state functional scans were acquired using an echo-planar imaging sequence with the following parameters: TR = 2000 ms, TE = 30 ms, FOV = 200 mm × 200 mm, FA = 90°, 33 axial slices, thickness/gap = 3.5 mm/0.6 mm, 64 × 64 matrix, and 240 volumes collected for 480 s.

All fMRI data were preprocessed using the Data Processing & Analysis for Brain Imaging (DPABI) software [27]. The following main steps were performed. First, the first 10 volumes were removed. The remaining 230 volumes were collected, and slice timing was corrected. Second, the head motion was corrected, and subjects with more than 2 mm of maximal translation and 2° of maximal rotation were excluded. Two HCs were excluded from further analysis due to excessive head motion. Third, the motion-corrected functional volumes were spatially normalized to the MNI space and resampled to an isotropic voxel size of 3 mm. Fourth, the processed images were smoothed with a 4 mm full width at half maximum (FWHM) Gaussian kernel, linearly detrended, and band-pass filtered (0.01-0.08 Hz). Fifth, the nuisance covariates, including white matter, 24 head motion parameters, and cerebrospinal fluid time course, were regressed out. Global signal regression (GSR) is a controversial issue in the resting-state fMRI preprocessing. Many researches clarified that the global signal contains some physiological signals, which are important and cannot be regressed out in the resting-state fMRI preprocessing [28, 29]. For this reason, we did not regress out the global signal in the current research. To verify whether the global signal has an impact on the current results, we reanalyzed the data with GSR. Finally, we scrubbed with a framewise displacement (FD) measure using a threshold of 0.2 together with one preceding and two subsequent volumes [30]. The mean FD for each participant was calculated, and no difference was observed between patients with OCD and HCs (Table 1).

### 2.3. Degree Analysis

Degree values represent the number of direct functional connections of a node with other nodes within the entire brain connectivity matrix. A correlation matrix is constructed by calculating the Pearson correlation coefficients of each voxel's time series to all other voxels' time series within a predefined gray matter mask. A threshold of 0.2 was used to remove the weak correlations when we constructed the voxel-voxel connectivity matrix [31]. Given the ambiguous explanation of negative correlations and detrimental effects of negative correlations on test-retest reliability, the present analyses were restricted to positive correlations by setting the negative correlations to 0 as described in the previous studies [17, 32, 33]. The degree value of a voxel was further computed as the sum of the connections at the individual level. Finally, the degree values were transformed into a *Z*-score map with the Fisher *Z* transformation in the whole brain voxel-wise for the improvement of normality.

### 2.4. SVM Analysis

SVM was conducted with the LIBSVM software (http://www.csie.ntu.edu.tw/cjlin/libsvm/). A “leave-one-out” cross-validation approach was used in verifying the performance of the SVM [34, 35]. One sample in each group was designated as the test sample, and the remaining samples were used as the training classifier. Then, the excluded subject pairs were used in testing the classifier's ability to reliably distinguish the groups (OCD/HCs). The step was repeated until the highest values for specificity and sensitivity were obtained [34, 35]. The global classification accuracy was obtained through the permutation testing, which was run 10,000 times for each sample (OCD/HCs).

### 2.5. Statistical Analysis

The clinical and demographic data of OCD and HCs were compared using two-sample *t*-tests and the chi-square test with SPSS Statistics 20.0 (IBM Corp., Armonk, NY, USA).

Two-sample *t*-tests were conducted using the DPABI software for the identification of difference in degree values between OCD and HCs. The potential influences of the mean framewise displacement (FD), age, gender, and HAMD and HAMA scores were reduced by using them as covariates. The threshold was set at *p* < 0.05 corrected by the Gaussian random field (GRF) theory for multiple comparisons.

Partial correlation analyses were performed between degree values showing between-group differences and clinical variables (i.e., Y-BOCS total score, obsessive thinking score, compulsive behavior score, HAMD, and HAMA scores). Gender, age, illness duration, and education were used as covariates in OCD. The significance level was *p* < 0.05 (Bonferroni corrected). Moreover, we conducted the whole-brain voxel-based correlations between degree values in the whole brain and clinical variables with gender, age, illness duration, and education as covariates in OCD.

## 3. Results

### 3.1. Demographics and Clinical Variables of Subjects

The demographics and clinical characteristics are presented in Table 1. Patients with OCD and HCs showed no significant difference in FD values, gender, age, or education. However, significant group differences in Y-BOCS, HAMD, and HAMA scores were found.

### 3.2. Group Differences in Degree Values

**Figure 1:**
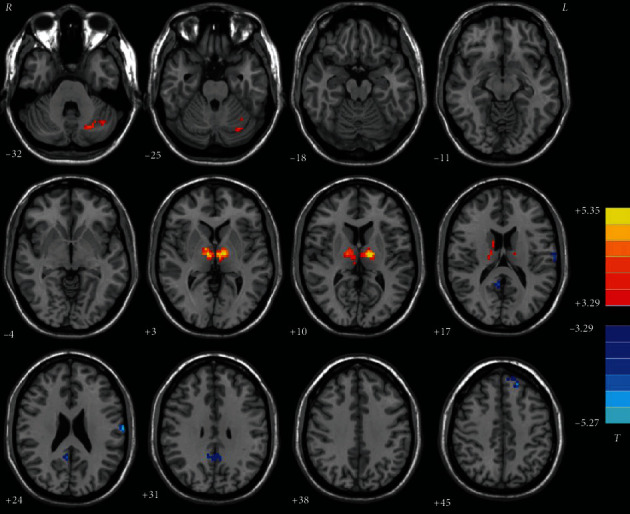
Brain regions with abnormal degree values in patients with OCD. *t* values from two-sample *t* tests with *p* < 0.05 (GRF corrected). Red denotes increased degree values; blue denotes decreased degree values. OCD = obsessive-compulsive disorder; GRF = Gaussian random field; L = left; R = right.

In comparison with HCs, patients with OCD had increased degree values in the left thalamus and left cerebellum Crus I and decreased degree values in the left DLPFC, right precuneus, and left postcentral gyrus (Table 2 and Figure 1). Furthermore, the results with GSR showed that patients with OCD had higher degree values in the left thalamus and lower degree values in the right precuneus (Table S1 and Figure S1 in Supplementary Materials).

### 3.3. Correlation Analysis

No relationship was observed between degree values showing between-group differences and clinical variables and dimensions in each clinical trait (i.e., Y-BOCS, HAMD, or HAMA) in OCD. There was also no correlation between any clusters and clinical variables in the patients with the whole-brain voxel-based correlation analyses.

### 3.4. SVM Results

**Figure 2:**
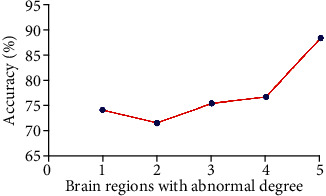
Accuracy of SVM using the five brain regions with abnormal degree values to separate OCD from HCs. The SVM result showed that the highest accuracy is 5. 1 = left cerebellum Crus I, 2 = right precuneus, 3 = left dorsolateral prefrontal cortex, 4 = left postcentral gyrus, and 5 = left thalamus. SVM = support vector machine; OCD = obsessive-compulsive disorder; HCs = health controls.

**Figure 3:**
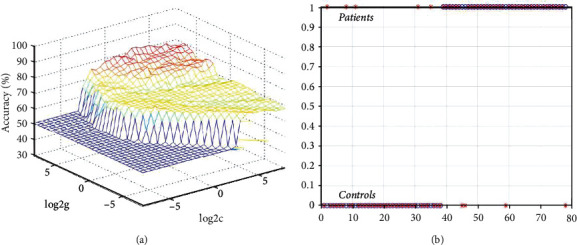
Visualization of SVM results for discriminating patients from controls using the degree values of the left thalamus. (a) 3D view of the classified accuracy with the best parameters. (b) Classified map of the degree values of the left thalamus. log2*c* and log2*g* mean the range and step size of the given parameters *c* and *g* (*c* and *g* are the parameters of the kernel functions in SVM training). The figure in (b) means the sensitivity and specificity of the SVM model. The horizontal axis conveys the predicted classification of each subject, and the vertical axis conveys the correct classification of each subject. SVM = support vector machine.

We used the altered degree values of the five brain regions for SVM classification. SVM results revealed that the accuracy of the left thalamus was the highest (Figure 2). Thus, the degree values in the left thalamus can be used in distinguishing OCD with an accuracy of 88.46% (69/78), a sensitivity of 87.50% (35/40), and a specificity of 89.47% (34/38) (Figure 3).

## 4. Discussion

The current study examined the whole-brain functional connectome in medication-free OCD at rest. Consistent with our hypothesis, patients with OCD showed altered degree values within the CSTC circuit (i.e., left DLPFC and left thalamus). In addition, the increased degree values in the left thalamus can be used in differentiating OCD from HCs. Moreover, OCD showed altered degree values outside the CSTC circuit (i.e., left cerebellum Crus I, right precuneus, and left postcentral gyrus).

The thalamus is a key region within the CSTC circuit, and thalamic-cortical dysconnectivity has been reported in OCD [36]. Increased gray matter volume and FC in the thalamus have been observed in OCD [8, 37–40]. Degree values of weighted networks are more resilient to FC disturbances, which are referred to as FC strength [15]. Previous studies have suggested that increased degree values are linked with increased FC strength by using the degree analysis to calculate the FC strength [31, 32]. In the current study, increased degree values in the left thalamus indicate increased functional strength between the thalamus and other brain regions at rest in OCD. Within the CSTC circuit, the thalamus is a gateway between the striatum and cortex, plays an important role in the integration of executive function and motor function, and controls the input and output of sensory information between the cortical motor areas and the basal ganglia [5, 36]. Increased functional strength in the thalamus is commonly explained as the compensatory reallocation of the thalamus for the activation of the connected brain areas [41, 42]. Therefore, increased functional strength in the thalamus may lead to excessive cortical information integration by activating the thalamic-cortical connectivity and may distort the subsequent behavioral selection process in OCD. Furthermore, the SVM classification is a binary classification algorithm that maximizes the boundary between classes in a high-dimensional space [43]. The current SVM results manifested that the increased degree in the left thalamus could be used as a future marker for OCD understanding-classification.

Within the CSTC circuit, we also observed decreased degree values in the left DLPFC, which is consistent with our previous findings in another independent OCD sample [44]. As an important brain region within the CSTC circuit, the DLPFC has been considered to be involved in the OCD pathophysiology [4, 44, 45]. Meanwhile, the DLPFC is the major component of the execution control network, which is related to executive functions during behavioral inhibition [46]. The decreased degree values of the left DLPFC indicate that the number of voxels located in the whole brain closely related to the left DLPFC decreased. Therefore, the ability of controlling intrusive thinking and repetitive behavior of OCD may reduce.

Apart from the CSTC circuit, the current study revealed increased degree values in the left cerebellum Crus I and decreased degree values in the right precuneus and the left postcentral gyrus at rest in OCD. The cerebellum is involved in the cognitive and affective process, which correlates with obsessive and ruminative behaviors [47]. Previous studies found increased FC in the cerebellum in OCD [48, 49], and our previous research reported increased cerebellar and default-mode network connectivity at rest in OCD [50]. Moreover, Sha et al. discovered that patients with OCD showed increased FC in the cerebello-thalamo-cortical networks [51]. Increased functional strength in the cerebellum may be involved in the compensatory response in the cognitive and affective process at rest in OCD [41, 42]. The precuneus is associated with self-awareness processing [52]. Reduced degree values may disrupt the balance of the precuneus and other brain regions and may result in difficulty in integrating inner thought and external events in OCD [53, 54]. As a key brain area of the somatosensory network, the postcentral gyrus plays an important role in sensory-motor integration and transmission [55]. Compared with HCs, the degree values of the left postcentral gyrus are reduced in patients with OCD in the current study. Previous researches also reported decreased ReHo and voxel-mirrored homotopic connectivity in the postcentral gyrus at rest in OCD [56, 57]. The decreased degree values of the left postcentral gyrus may reduce the efficiency of information transmission within the sensory-motor pathway, therefore contributing to the repetitive and intrusive thoughts and behaviors in patients with OCD [58].

Previous studies revealed that altered degree values were mainly observed in the CSTC circuit (i.e., OFC and basal ganglia) and emotional modulation network (i.e., ventral frontal cortex and amygdala) in OCD [21, 22, 59]. Consistent with the previous research, the present study discovered altered degree values within the CSTC circuit (i.e., left DLPFC and left thalamus) but failed to discover altered degree values in the emotional modulation network at rest in OCD. Different sample sizes, clinical symptoms, medication status, data analysis, and different OCD subtypes may account for these inconsistencies [59–62]. Moreover, inconsistent with our hypothesis, the current study did not find any relationship between degree values showing between-group differences and clinical variables in OCD. We speculated that the abnormal degree values were possibly trait changes for OCD [63].

GSR is a controversial issue in the resting-state fMRI preprocessing. Many researches clarified that the global signal contains some physiological signals, which are important and cannot be regressed out in the resting-state fMRI preprocessing [28, 29]. For this reason, we did not regress out the global signal in the current research. Furthermore, different from the results without GSR, the results with GSR showed that patients with OCD had higher degree values in the left thalamus and lower degree values in the right precuneus, suggesting that GSR has an impact on the resting-state fMRI results [64].

Several limitations must be considered. First, 22 patients had a history of psychotropic medication, and the current results may be affected by psychotropic medication. Second, we only discovered some brain regions showing altered degree values at baseline in OCD. The effects of medication, psychotherapy, and physical therapy on changes in degree values in OCD should be investigated in future studies. Third, the present study did not collect cognitive and behavioral information. Fourth, the SVM results were not tested in another independent sample, presumably leading to overfitting and optimistic results. The leave-one-out approach was used to construct the model and to perform the SVM analysis due to the small sample size, which again could cause the overfitting issue. Fifth, a previous study has found that the SVM analysis needs at least 200 subjects to observe the reliable results [65]. The sample size of the current research was relatively small, and the power of SVM classification was limited, which was insufficient to draw strong conclusions based on the identified anomalies. Therefore, further researches are needed to use an alternate atlas for parcellation in order to make wiser conclusions in a small dataset [66]. Sixth, like degree analysis, the network homogeneity (NH) method can be used as an important index for evaluating the FC strength [67]. In a previous research, we used the NH method to investigate the FC strength within the default-mode network (DMN) in the same OCD sample [68]. Some similar results (i.e., decreased FC strength in the right PCC/PCu) were found between these two researches, suggesting that the current results can be reproduced to a certain extent. However, due to small sample size, the results (degree classifying neural areas) should be taken with caution. Finally, we did not divide OCD into different subtypes according to clinical symptoms. Future researches should strictly control the heterogeneity of OCD samples.

In conclusion, the current study discovered altered degree values within and outside the CSTC circuit at rest in OCD. The increased degree values of the left thalamus could be used as a future marker for OCD understanding-classification.

## Figures and Tables

**Table 1:** Demographic and clinical characteristics of participants.

	OCD patients (*n* = 40)	HCs (*n* = 38)	*X*^2^/*t*	*p*
Age (years)	27.28 ± 8.16	27.18 ± 8.33	0.05	0.71
Sex (male/female)	27/13	25/13	0.026	0.87∗
Education (years)	13.40 ± 2.87	13.74 ± 3.03	-0.50	0.83
Illness duration (months)	66.68 ± 75.54			
Y-BOCS total score	24.90 ± 5.73	1.13 ± 0.88	25.27	<0.001
Y-BOCS obsessive thinking	12.85 ± 4.25	0.37 ± 0.49	17.98	<0.001
Y-BOCS compulsive behavior	12.05 ± 4.62	0.74 ± 0.72	14.92	<0.001
HAMD	8.05 ± 4.40	1.45 ± 0.95	9.04	<0.001
HAMA	10.83 ± 6.55	1.16 ± 1.00	9.00	<0.001
FD	0.04 ± 0.02	0.03 ± 0.01	1.25	0.13
Time points scrubbed out	1.13 ± 2.256	1.00 ± 2.418	0.25	0.95

OCD = obsessive-compulsive disorder; HCs = health controls; Y-BOCS = Yale-Brown Obsessive-Compulsive Scale; HAMD = 17-item Hamilton Depression Rating Scale; HAMA = Hamilton Anxiety Rating Scale; FD = framewise displacement. Variables of age, education, Y-BOCS total score, subscale score, HAMD score, HAMA score, and FD were tested by two-sample *t*-tests, and the results were indicated by *t* values. Categorical data such as gender was tested using the chi-square test, and the result was indicated by *Χ*^2^.

**Table 2:** Regions with abnormal degree values in the patients with OCD.

Cluster location	Peak (MNI)			Number of voxels	*t* value
	*x*	*y*	*z*		
Left thalamus	-12	-12	9	198	5.3545
Left cerebellum Crus I	-30	-72	-27	64	4.7578
Left DLPFC	-18	42	45	25	-4.7994
Right precuneus	6	-51	21	57	-4.7865
Left postcentral gyrus	-66	-15	21	25	-5.2707

All effects survived a voxel-wise statistical threshold (*p* < 0.05) after Gaussian random field (GRF) correction for multiple comparisons (voxel significance: *p* < 0.001, cluster significance: *p* < 0.05). OCD = obsessive-compulsive disorder; MNI = Montreal Neurological Institute; DLPFC = dorsolateral prefrontal cortex.

## Data Availability

Our data may be available upon reasonable request. Please contact lipingchxyy@163.com for details.

## Supplementary Materials

Supplementary Materials Table S1: abnormal degree values in the patients with OCD (GSR removed). Figure S1: abnormal degree values in the patients with OCD (GSR removed). *t* values from two-sample *t*-tests with *p* < 0.05 (GRF corrected). Red denotes increased degree values; blue denotes decreased degree values. OCD = obsessive-compulsive disorder; GSR = global signal regression; L = left; R = right.
