# Supplementary material for: Altered Functional Connectivity Strength at Rest in Medication-Free Obsessive-Compulsive Disorder
Source: Neural Plast. 2021 Sep 8;2021:3741104. doi: 10.1155/2021/3741104 (PMC8443365; doi:10.1155/2021/3741104)
Supplement: Supplementary Materials — Table S1: abnormal degree values in the patients with OCD (GSR removed). Figure S1: abnormal degree values in the patients with OCD (GSR removed). t values from two-sample t-tests with p < 0.05 (GRF corrected). Red denotes increased degree values; blue denotes decreased degree values. OCD = obsessive-compulsive disorder; GSR = global signal regression; L = left; R = right. [file 3741104.f1.docx]

**TABLE S1:** Abnormal degree values in the patients with OCD (GSR removed)

| Cluster location | Peak (MNI) | | | Number of voxels | *T* value |
| --- | --- | --- | --- | --- | --- |
|  | x | y | z |  |  |
| left Thalamus | -12 | -9 | 3 | 116 | 4.8427 |
| Right Precuneus | 6 | -51 | 21 | 107 | -6.0219 |

All effects survived a voxel-wise statistical threshold (*p*<0.05) after Gaussian random field (GRF) correction for multiple comparisons (voxel significance: *p*<0.001, cluster significance: *p*<0.05). The mean framewise displacement (FD), age, gender, and HAMD and HAMA scores were used as covariates. OCD = obsessive-compulsive disorder; GSR = global signal regression; MNI = Montreal Neurological Institute.


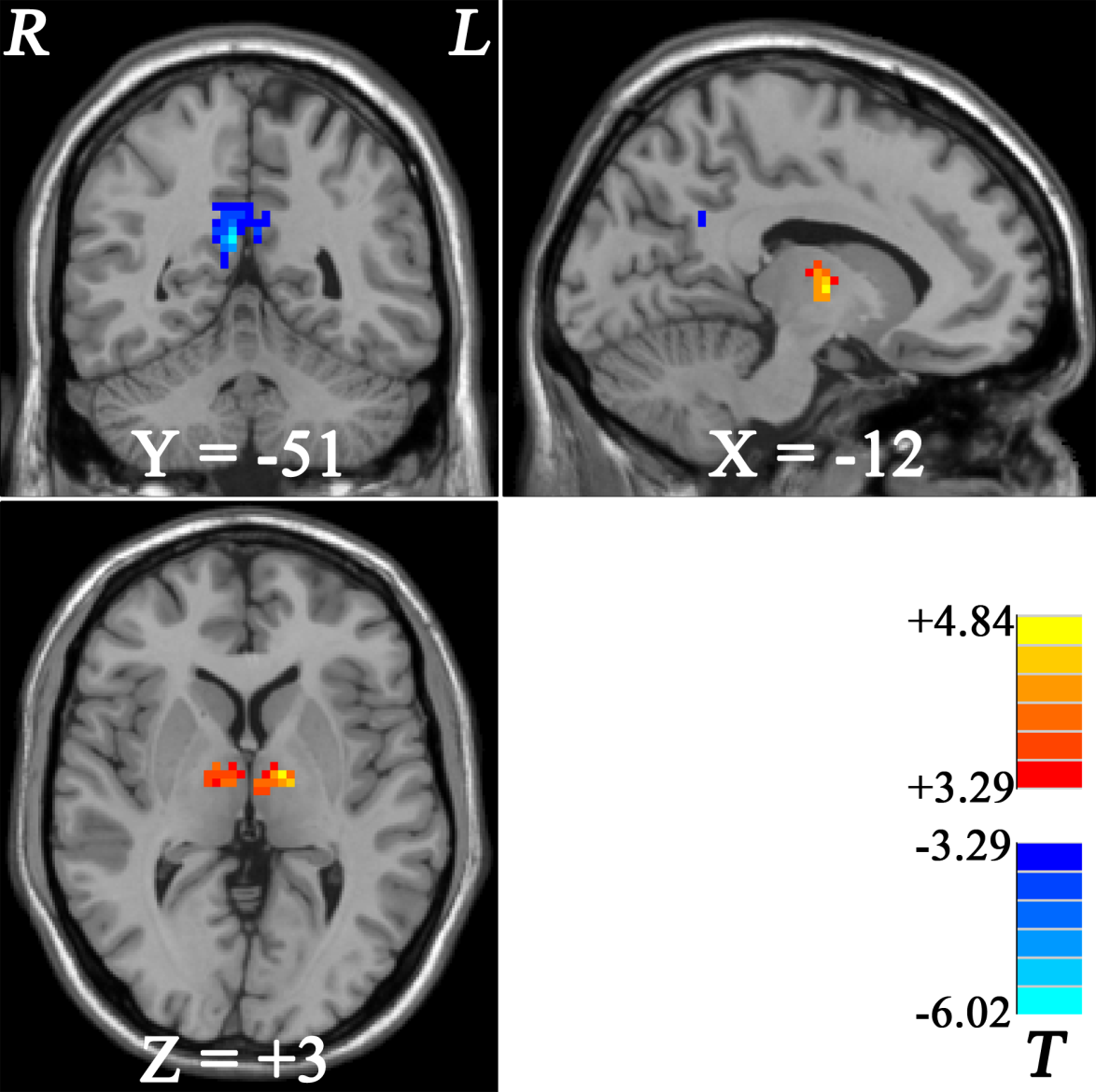


**Figure S1:** Abnormal degree values in the patients with OCD (GSR removed).

T values from two-sample *t* tests with *p* < 0.05 (GRF corrected). Red denotes increased degree values; blue denotes decreased degree values. OCD = obsessive-compulsive disorder; GSR = global signal regression; L = left; R = right.
